# Ideal T1 laminar screw fixation based on computed tomography morphometry

**DOI:** 10.1186/s12891-017-1604-3

**Published:** 2017-06-02

**Authors:** Xiao-Bo Wang, Xin Zheng, Hou-Qing Long, Wen-Li Chen, Xing Cheng, Yang-Liang Huang, Jing-Hui Xu

**Affiliations:** 1 Department of Spine Surgery, The First Affiliated Hospital of Sun Yat-sen University, Guangzhou, 510080 People’s Republic of China; 2 Department of Joint Surgery, The Affiliated Hospital of Xuzhou Medical University, Xuzhou, 221000 People’s Republic of China; 3 Department of Neurosurgery, The First Affiliated Hospital of Sun Yat-sen University, Guangzhou, 510080 People’s Republic of China; 4 NO.183 Huangpu East Road, Guangzhou, 510000 Guangdong People’s Republic of China

**Keywords:** Laminar screw, Upper thoracic spine, CT scan, Radiographic parameters

## Abstract

**Background:**

The application of laminar screws is an alternative fixation for the first thoracic vertebra (T1). This paper is to determine the anatomical characteristics for adequate laminar screw fixation, and present a modified method of sagittal reconstruction of T1 to provide more accurate measurements.

**Methods:**

Computed tomography (CT) images of 62 patients (32 males, 30 females) were used for the analysis. The following parameters of the T-1 lamina were measured using Mimics software: lamina length, axis angle, minimal outer cortical width, cancellous width, minimal outer cortical height, cancellous height, and spinous process height. Right or left modified sagittal reconstructions (parallel to right or left screws) were innovatively used for measurement.

**Results:**

There were no significant differences between the left and right sides for each measurement performed (*P* > 0.05), but significant differences were detected between males and females (*P* < 0.05). The mean length of the T1 lamina was 32.8 mm of the T1 minimal outer cortical width was 7.4 mm, and 3.8% of males had a minimal outer cortical width < 5 mm, while 8.6% of females had a minimal outer cortical width < 5 mm. The mean minimal outer cortical height was 10.8 mm, and 1.9% of males had a minimal outer cortical height < 9 mm, while 7.7% of females had a minimal outer cortical height < 9 mm.

**Conclusion:**

This study suggests there are no anatomical limitations for T1 laminar screw placement in most people. The modified sagittal reconstruction method described allows for easy and precise measurement to aid in the insertion of laminar screws in T1, and gives good visualization of laminar screw insertion direction.

## Background

There are many fixation options for the surgical treatment of cervicothoracic junction (CTJ) disease, deformity, tumors and spinal canal stenosis occurring in the lower cervical and upper thoracic region. Pedicle screws fixation is commonly used for fixation in patients with CTJ disease, but it is a challenge for many surgeons. The C7-T1 segment is a transition from the mobile, lordotic cervical spine to the relatively rigid, kyphotic thoracic spine [1–3]. Because of the complex biomechanics of this region, there is a high possibility of construct failure when performing fixation. Furthermore, the anatomical features also make internal fixation difficult.

Stanescu et al. [4] reported that the T1pedicle height is the shortest in the thoracic spine. In another study, Privitera et al. [5] implanted 1042 pedicle screws in T1-T3, and reported that 8.3% were misplaced, and the highest misplacement rate was at T1. Previous studies have also verified that the superior and inferior nerve roots of T1 and T2 are close at their exit, which make them easy to injury during pedicle screws insertion [6]. For these reasons, classical pedicle screw fixation, which is the gold standard for thoracic and lumbar spinal instrumentation, is difficult to perform in this region, especially for T1.

Laminar screws were initially developed for lumbar spine fixation [7]. Wright et al. [8] used laminar screws for C2 fixation, and considered it a safe alternative to pedicle fixation for avoiding vertebral artery injury. Compared to the pedicle screw, laminar screw fixation has several advantages including lamina visualization during surgery, and a trajectory that is posterior to the spinal cord and nerve roots. The feasibility of translaminar screw fixation has been demonstrated by clinical trials, and biomechanical and anatomical studies [9–11].

Laminar screws have better insertional torque and screw pullout strength than pedicle screws at T1/T2 [12]. However, radiographic measurements related to the insertion of laminar screws are limited, especially for the minimal laminar height required [13–15]. Hu et al. [13] suggested that the bilateral heights of the middle 1/3 narrowest lamina should be considered the bilateral minimal outer cortical heights. Other studies, however, did not describe how the minimal outer cortical height was measured, and the sagittal reconstructions did not provide visualization of the whole vertical section of lamina [14, 15]. Thus, data reported of the minimal outer cortical height in these studies may not be accurate.

In this study, computed tomography (CT) was used to determine the imaging parameters characteristics of T1 lamina in healthy Han adults. A modified sagittal reconstruction was innovatively created using a line which was vertical and paralleled to the ideal laminar screw trajectory. In this reconstruction, the laminar vertical section was visible, and the minimal outer cortical and cancellous heights could be measured easily and accurately. Thus, the purposes of this study were to determine the anatomical characteristics for adequate laminar screw fixation in the first thoracic vertebra (T1), and present a modified method of sagittal reconstruction of T1 to provide more accurate measurements.

## Methods

### Patients

Sixty-six patients (23 with pneumonia, 3 with lung bullae, 16 with lung tumors, and 24 with chest trauma) who underwent chest CT scans from November 2014 to September 2015, were included in this study. None of the patients had thoracic spine degenerative disease, fractures, osteoporosis, or tumors that influenced T1. The age of the 66 patients (33 males, 33 females) ranged from 20 to 70 years. The average age was 50.6 ± 13.3 years (52.5 ± 13.4 years for males and 48.3 ± 12.8 years for females). The average age of males and females was not significantly different (*P* < 0.05). The informed consent was waived by the Medical Ethics Committee as this was a retrospective study utilizing the data and images with all patient identifying information removed.

### CT scanning and laminar measurements

**Fig. 1:**
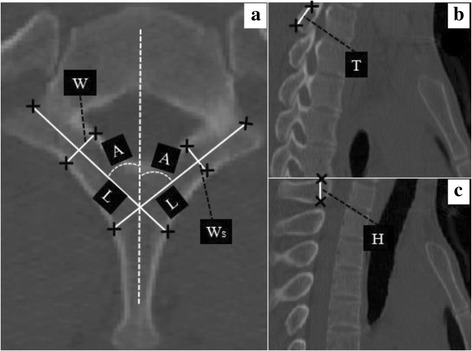
Measurements of T1 on a transverse computed tomography (CT). **a** L: length based on the ideal laminar screw trajectory. A: the axial angle between the ideal laminar screw trajectory and the midline. W: the minimal outer cortical width. Ws: the minimal cancellous width. **b** Minimal outer cortical width (T) measured in the sagittal reconstruction at the middle 1/3 narrowest lamina. **c** The spinous process height (H) obtained at the site of the cross-point of the bilateral ideal laminar screw trajectory in the coronal plane

**Fig. 2:**
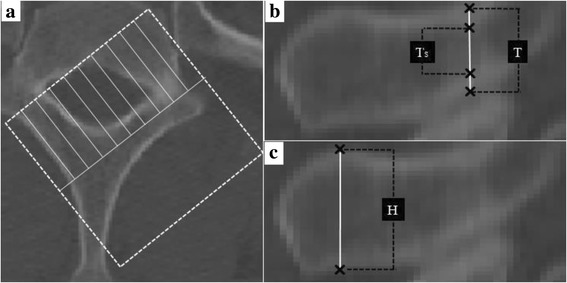
**a** Process for creating the modified sagittal reconstruction. **b** Minimal outer cortical (T) and cancellous heights (Ts) obtained at the narrowest point of the lamina in the modified sagittal reconstruction. **c** Spinous process height (H) was measured at the cross-point of the bilateral ideal laminar screw placement in the modified sagittal reconstruction

Patients were scanned using a Light Speed 16 Pro spiral CT scanner (GE, Connecticut, USA) with 0.625 mm CT slices at 300 mA and 120 kV. DCM file format images were imported into Mimics software (Materialise, Mimics Research 17.0 × 64). A reference coordinate system was established to measure the geometric features of the 3-dimansional (3D) reconstructed T1 vertebra. A line that was determined by a proficient surgeon based on the anatomical axis of the individual lamina to imitate the ideal laminar screw trajectory was defined as the X-axis. The line bisecting the spinal canal in the anteroposterior direction was set as the Z-axis. The line that passed through the cross-point of the bilateral X-axis, and was perpendicular to the X-axis and Z-axis, was set as the Y-axis. Traditional transverse and sagittal reconstructions of T1 were generated (Fig. 1). A reconstruction that was created along the Y-axis was defined as the modified sagittal reconstruction (Fig. 2a).

In the transverse reconstruction, the T1 laminar length (L) was measured from the junction of the lateral mass and lamina to the contralateral outer cortex of the spinous process on the X-axis. The cross-angle between the X-axis and Z-axis was defined as axis angle (A). The minimal outer cortical width (W), and minimal cancellous width (W_s_) were recorded at the narrowest point of the lamina along the X-axis (Fig. 1a). In the sagittal reconstruction, the minimal outer cortical (Fig. 1b) and cancellous heights were measured as previously described [13]. The spinous process height was obtained at the site of the cross-point of the bilateral X-axis in the coronal plane (Fig. 1c).

In the modified sagittal reconstruction, the vertical section of the lamina was visible, and the minimal outer cortical and cancellous heights were measured at the thinnest portion of the lamina along the X-axis (Fig. 2b). The height of spinous process was measured at the cross-point of bilateral X-axis (Fig. 2c). Theoretically, the heights of spinous processes measured in the left and right modified coronal reconstructions are the same, and thus can be used as a reference to test the accuracy in the 2 reconstructions. If the left and right spinous process heights were significantly different, the reconstruction and measurements were repeated. If differences were again found, the data of that person was excluded from the analysis.

### Inter- and intra-observer measurement variability

To examine the inter- and intra-observer variability in the measurement, the parameters of 10 patients were measured by 2 readers (Wang XB, Zheng X) on 2 different occasions.

## Statistical analysis

Statistical analysis was performed using SPSS 17.0 for Windows. Pearson’s rank correlation coefficients were used to assess measurement variability. The paired student t-test was used to detect differences between the left and right sides, and between the sagittal and the modified sagittal reconstructions. The 2-sample student t-test was used to compare data between males and females. The threshold for statistical significance was defined as a value of *P* < 0.05.

## Results

### Patients

CT imaging showed that there were significant differences in the right and left spinous process heights in 1 male and 3 females, thus, the data of these patients were excluded. As a results, the CT images of 62 patients (32 males, 30 females) were included in the analysis. Patient data are presented in Tables 1, 2 and 3.

**Table 1:** Measurements in T1 stratified by side of screw placement and patient’s sex^a^

Measurements	Total	Male	Female	t	P	Left	Right	t	P
L (mm)	32.8 ± 3.2	34.8 ± 2.8	30.7 ± 2.1	9.405	*P* < 0.05	32.6 ± 3.3	33.0 ± 3.3	-1.971	*P* > 0.05
W (mm)	7.4 ± 1.3	8.1 ± 1.2	6.7 ± .1.1	6.811	*P* < 0.05	7.4 ± 1.3	7.4 ± 1.3	-0.128	*P* > 0.05
Ws (mm)	4.4 ± 0.9	4.8 ± 0.9	3.8 ± 0.6	7.101	*P* < 0.05	4.3 ± 0.9	4.4 ± 0.9	-1.772	*P* > 0.05
A (°)	48.6 ± 3.5	47.2 ± 3.2	50.1 ± 3.2	-5.044	*P* < 0.05	48.7 ± 3.2	48.5 ± 3.9	0.243	*P* > 0.05
T (mm)	10.8 ± 2.1	11.9 ± 2.0	9.6 ± 1.5	7.452	*P* < 0.05	10.7 ± 2.2	10.8 ± 2.0	-0.276	*P* > 0.05
MT (mm)	10.1 ± 2.3	11.3 ± 2.2	8.8 ± 1.5	7.293	*P* < 0.05	9.9 ± 2.3	10.2 ± 2.2	-0.572	*P* > 0.05
Ts (mm)	6.1 ± 1.6	6.6 ± 1.7	5.5 ± 1.3	3.772	*P* < 0.05	6.0 ± 1.6	6.1 ± 1.7	-0.998	*P* > 0.05
MTs (mm)	5.8 ± 1.7	6.3 ± 1.8	5.2 ± 1.3	3.961	*P* < 0.05	5.6 ± 1.6	5.9 ± 1.7	-1.399	*P* > 0.05
H (mm)	13.5 ± 1.4	14.0 ± 1.3	12.8 ± 1.2	5.184	*P* < 0.05	13.4 ± 1.4	13.5 ± 1.4	-1.447	*P* > 0.05
MH (mm)	13.6 ± 1.6	14.1 ± 1.5	13.0 ± 1.4	4.222	*P* < 0.05	13.6 ± 1.6	13.6 ± 1.6	-1.616	*P* > 0.05

^a^The laminar screw length (L), the minimal outer cortical width (W), the minimal cancellous width (W_s_), the minimal outer cortical height (T, MT), the minimal cancellous height (T_s_, MT_s_), the axis angle (A) and the spinous process height (H, MH) in T1 are reported as $\overset{-}{\mathrm{X}}$ ±S. T, T_s_, and H were measured in the sagittal reconstruction, while the MT, MT_s_, and MH were measured in the modified sagittal reconstruction.

**Table 2:** The minimal outer cortical height (T), the minimal cancellous height (T_s_) and the spinous process height (H) stratified by sagittal and modified sagittal reconstructions^a^

Measurements	sagittal	Modified sagittal	t	P
T	10.8 ± 2.1	10.1 ± 1.7	15.553	*P* < 0.05
T_s_	6.1 ± 1.6	5.7 ± 1.7	13.587	*P* < 0.05
H	13.5 ± 2.3	13.6 ± 1.6	-1.256	*P* > 0.05

^a^The three parameters were report as $\overset{-}{\mathrm{X}}$ ±S.

**Table 3:** Comparison of the T1 laminar morphology with the literature data^a^

Measurements	Groups	Present study	Modified sagittal reconstruction	Patel, USA^[b]^	Molina, USA^[c]^	Hu, China^[d]^	Kretzer, USA^[e]^
Patient’s age		20-70 years old	20-70 years old	8-18 years old	2-16 years old	19-78 years old	>18 years old
L	Total	32.8 ± 3.2		29.9 ± 4.1		30.8 ± 3.4	33.4 ± 3.6
	Males	34.8 ± 2.7		30.6 ± 4.2	31.9	32.0 ± 3.2	34.7 ± 3.6
	Females	30.7 ± 2.1		29.1 ± 4.0	28.9	29.6 ± 3.1	32.0 ± 3.0
W	Total	7.4 ± 1.3		6.5 ± 1.3		6.2 ± 1.0	6.6 ± 1.1
	Males	8.1 ± 1.2		6.6 ± 1.4	5.7	6.5 ± 0.8	6.9 ± 1.1
	Females	6.7 ± 1.1		6.4 ± 1.2	4.8	5.9 ± 1.0	6.3 ± 1.0
Ws	Total	4.4 ± 0.9				3.4 ± 1.0	3.7 ± 1.0
	Males	4.8 ± 0.9			2.4	3.6 ± 1.1	4.0 ± 1.0
	Females	3.8 ± 0.6			2.0	3.2 ± 0.9	3.3 ± 0.9
A	Total	48.6 ± 3.5		47 ± 4		99.9 ± 3.6	49.2 ± 3.7
	Males	47.2 ± 3.2		47 ± 4	50.0	99.6 ± 4.2	49.1 ± 4.0
	Females	50.1 ± 3.2		46 ± 4	50.0	100.2 ± 3.1	49.3 ± 3.4
T	Total	10.8 ± 2.1	10.1 ± 2.3	12.3 ± 3.4		16.9 ± 1.3	
	Males	11.9 ± 2.0	11.3 ± 2.2	11.8 ± 3.4		17.6 ± 1.3	
	Females	9.6 ± 1.5	8.8 ± 1.5	12.8 ± 3.3		16.4 ± 1.0	
Ts	Total	6.1 ± 1.6	5.7 ± 1.7				
	Males	6.6 ± 1.7	6.3 ± 1.8		11.4		
	Females	5.5 ± 1.3	5.2 ± 1.3		10.3		
H	Total	13.5 ± 1.4	13.6 ± 1.6				
	Males	14.0 ± 1.3	14.1 ± 1.5				
	Females	12.8 ± 1.2	13.0 ± 1.4				

^a^The translaminar screw length (L), the minimal outer cortical width (W), the minimal cancellous width (W_s_), the minimal outer cortical height (T), the minimal cancellous height (T_s_), the axis angle (A) and the spinous process height (H) in T1 are reported as $\overset{-}{\mathrm{X}}$ ±S.

[^b^] Patel AJ, Cherian J, Fulkerson DH, Fox BD, Chern JJ, Whitehead WE, Curry DJ, Luerssen TG, Jea A (2011) Computed tomography morphometric analysis for translaminar screw fixation in the upper thoracic spine of the pediatric population. Journal of neurosurgery Pediatrics 7 (4):383-388.

[^c^] Molina C, Sciubba DM, Chaput C, Tortolani PJ, Jallo GI, Kretzer RM (2012) A computed tomography-based feasibility study of translaminar screw placement in the pediatric thoracic spine. Journal of neurosurgery Pediatrics 9 (1):27-34.

[^d^] Hu QF, Xu RM, Pan H, Zhou H, Lei W (2015) Translaminar Screw Fixation in the Upper Thoracic Spine: Computed Tomography-Based Quantitative Laminar Analysis and Feasibility Study of Translaminar Virtual Screw Placement. Cell biochemistry and biophysics 2015, 73(1): 191-198.

[^e^] Kretzer RM, Chaput C, Sciubba DM, Garonzik IM, Jallo GI, McAfee PC, Cunningham BW, Tortolani PJ (2010) A computed tomography-based feasibility study of translaminar screw fixation in the upper thoracic spine. Journal of neurosurgery Spine 12 (3):286-292.

### Length (L) and angle (A)

The mean length of the T1 lamina was 32.8 ± 3.2 mm in (range from 26.1 to 39.7 mm) for the total population. The mean length in males was significantly greater (3 mm) than in females (*P* < 0.05). The mean length of the right T1 lamina was greater than that of the left T1 lamina; however, the difference was not statistically significant (*P* > 0.05). The ideal laminar screw trajectory was 48.5 ± 3.5° in relation to the line bisecting the spinal canal. No difference of ideal trajectory was noted between the right and left side (*P* > 0.05), but a significant difference was detected based on sex (*P* < 0.05).

### Minimal outer cortical width (W) and minimal cancellous width (W_s_)

The average minimal outer cortical width was 7.4 ± 1.3 mm (range, 4.2-9.4 mm), and the mean minimal cancellous width was 4.4 ± 0.9 mm (range, 2.9-6.8 mm). There was no significant difference in the minimal laminar width between the right and left sides (*P* > 0.05). The minimal outer cortical laminar width of males was significantly greater (by 1.4 mm) than that in females, as was the minimal cancellous width (by 1.0 mm) (both, *P* < 0.05) (Table 1). When the minimal laminar cortical width was compared with potential screw diameter, laminar screws that were 4.0 mm in diameter with 1.0 mm clearance could be used in 89.5% of patients. With respect to minimal cancellous width, it was <4 mm in 54.0% of patients.

### Minimal outer cortical height (T) and cancellous height (T_s_)

In the sagittal reconstruction, the mean minimal outer cortical and cancellous heights were 10.8 ± 2.1 mm (range, 5.7-16.5 mm) and 6.1 ± 1.6 mm (range, 2.2-11.0 mm), respectively. There was no difference between the left and right for the 2 parameters (*P* > 0.05). The mean minimal outer cortical height of males was 2.3 mm greater than that of females (*P* < 0.05) (Table 1). In addition, the mean minimal outer cortical height was <9 mm in 2 sides of males (3.1%) and 21 sides of females (35%), accounting for 18.5% of the total. The mean minimal cancellous height was 6.1 ± 1.6 mm for 18.5% shorter than 9 mm in 2 sides of men and 21 sides of women (*P* < 0.05).

In the modified sagittal reconstruction, the mean minimal outer cortical and cancellous heights were 10.1 ± 1.7 mm (range, 4.5-15.8 mm) and 5.7 ± 1.7 mm (range, 1.6-10.8 mm), respectively. There were not differences in these parameters between the left and right sides (both, *P* > 0.05); however, there was a significant difference between males and females (*P* < 0.05) (Table 1). A minimal outer cortical height < 9 mm was present in 10 sides of males (15.6%) and 34 sides of females (56.7%), accounting for 35.5% of the total. The minimal outer cortical height in the modified sagittal reconstruction was significant less than that in the sagittal reconstruction (*P* < 0.05), as was the minimal cancellous height (*P* < 0.05) (Table 2).

### Spinous process height (H)

Spinous process heights were measured in every patient, and no anatomical variants <9 mm were noted. No significant difference was detected between the left and right side (*P* > 0.05), but a significant difference was found between males and females (*P* < 0.05) (Table 1). No significant difference in spinous process heights were found between the sagittal and modified sagittal reconstruction (all, *P* > 0.05) (Table 2).

### Inter- and intra-observer variability

The Pearson correlation coefficients for interobserver repeat measurements ranged from 0.96 to 0.99, and the correlation coefficients for intraobserver repeat measurements ranged from 0.94 to 0.99. All correlations were statistically significant, and the level of measurement reliability was excellent.

## Discussion

Although pedicle screw fixation has become a very common method of spinal fixation, there are drawbacks that include pseudoarthritis, adjacent segment degeneration, and neurological impairment [16]. Laminar screws have been shown to have a lower incidence of vertebral artery and nerve root injury as compared to pedicle screws [8, 17, 18]. When pedicle screws are used in patients with marked osteoporosis, screw loosening is frequent. Cardoso et al. [19] demonstrated that the anti-pullout strength of pedicle screws is correlated with bone mineral density in the upper thoracic spine, while this correlation is not present with laminar screws. Therefore, laminar screws may provide stronger fixation in patients with osteoporosis.

To define the anatomical shape of the T1 lamina for inserting laminar screws, a comprehensive understanding of the bone morphology is essential. However, current morphological studies of the lamina are limited [13, 14, 20]. The morphology of the vertical section of the lamina is not visualized in many studies, and using transverse reconstructions to determine the narrowest laminar heights can provide inaccurate results. The current study described an improved method for laminar sagittal reconstruction that can provide the morphology of the laminar vertical section (Fig.2b). The minimal height could be easily and precisely measured in this reconstruction, making it clinically valuable. The detailed anatomical data of T1 obtained from Chinese Han may be useful for inserting laminar screws in this population. However, the anatomical structure of T1 varies by ethnicity and region, and thus the data may not be generalizable to other populations and explain differences between the results of this study and that of Hu et al. [13].

The mean minimal outer cortical and cancellous heights in the sagittal reconstruction were greater than that in the modified sagittal reconstruction. This proved that the site where the lamina is thinnest in the transverse reconstruction was not the same as in the sagittal reconstruction. Although the difference of minimal outer cortical height between the sagittal and modified sagittal reconstructions is minimal, using data from the modified reconstruction can increase the likelihood that screws will be inserted into cortical bone, and thus have greater biomechanical strength. Furthermore, the point of smallest minimal outer cortical height could be identified more precisely in the modified sagittal reconstruction. The sagittal axis for the screw insertion should be based on this point. In this study no anatomical variants of T1 laminar heights were found that would affect the insertion of bilateral laminar screws. In addition, we tentatively put forward that the statistical differences may be great in other computed tomography morphometric studies when the reconstruction is created along the center line of the tissues. Unfortunately, this problem has not been well explained in previous morphometric studies. In many researches, parameters were measured in the transverse and parasagittal reconstruction which could probably result in serious measuring error.

The minimal outer cortical width is the most important factor for the placement of laminar screws. In the current study population, the mean minimal outer cortical width was 7.4 ± 1.3 mm. Many authors consider that the minimal outer cortical width should be >5 mm to safely hold a 4 mm laminar screw with 1.0 mm clearance [15, 21, 22]. Therefore, in this study the width was too narrow for screw placement in 6.3% of the patients. Hu et al. [13] described similar results in the Chinese adult population (Table 3). Molina et al. [23] reported that the average minimal outer cortical width was 5.7 mm for males and 4.8 mm for females, and in 76% of males the minimal outer cortical width was >5 mm, and was >5 mm in 65% of females. However, in Molina’s series patient age ranged from 2 to 16 years old (Table 3).

If minimal cancellous width and height requirements are met, laminar screws have excellent biomechanical strength [12]. The percentage of patients in this study in whom the minimal cancellous width was <4 mm was 54.0%. That means the purchase of laminar screws could achieve the cortex for most people, providing the screws with greater inline pullout strength and insertional torque. This superiority offsets the deficiency that laminar screws could not provide 3-column fixation [24].

Lamina length does not affect the decision to use laminar screws, but is helpful for selecting the optimal screw length. Hu et al. [13] investigated T1 anatomical morphology in 40 patients, and found that the mean maximal length for laminar screws was 30.8 mm, and ranged from 23.1 mm to 38.6 mm (Table 3). The results of our study were similar to that of Hu et al., but the choice of laminar screw length was significantly affected by sex.

The axis angle describes the optimal inclination in relation to the midline spinous process for proper insertion of laminar screws. Ventral cortical wall violation could lead cerebrospinal fluid leakage or spinal cord injury, and inserting the screw at the ideal inclination could reduce these risks. Our results showed that the ideal laminar screw trajectory relative to the spinous process was 48.6 ± 3.5°, and there was a significant difference between males and females. However, Kretzer et al. [24] reported there was no difference in axis angle between males and females.

There are several limitations of this study that should be considered. Although CT imaging provides data for determining the ideal screw length and diameter, 2D data should be translated to a 3D reconstruction to improve the accuracy of screw insertion, and correlate insertion with bone surface features. Pedicle morphological parameters were not measured, and differences between the lamina and pedicles were no examined.

## Conclusion

There are no anatomical limitations for T1 laminar screw placement in most people based on CT data collected in this study. The modified sagittal reconstruction method described allows for easy and precise measurement to aid in the insertion of laminar screws in T1, and gives good visualization of laminar screw insertion direction. Parameters measured in the reconstruction created along the center line could be more credible.

## Abbreviations

3D: 3-dimansional
CT: Computed tomography
CTJ: Cervicothoracic junction
T1: First thoracic vertebra
